# Duration discrimination: A diffusion decision modeling approach

**DOI:** 10.3758/s13414-022-02604-1

**Published:** 2023-01-23

**Authors:** Lukas Schumacher, Andreas Voss

**Affiliations:** Institut of Psychology, Department of Quantitative Research Methods, Heidelberg University, Hauptstrasse 47-51, 69117 Heidelberg, Germany

**Keywords:** Diffusion decision model, Duration discrimination, Context effects

## Abstract

The human ability to discriminate the duration of two subsequently presented stimuli is often studied with tasks that involve a comparison between a standard stimulus (with fixed duration) and comparison stimuli (with varying durations). The performance in such tasks is influenced by the presentation order of these successively presented stimuli. The so-called Type A effect refers to the impact of presentation order on the point of subjective equality. The Type B effect describes effects of presentation order on the just-noticeable-difference. Cognitive models that account for these context effects assume that participants’ duration estimation is influenced by the history of previously encountered stimuli. For example, the internal reference model assumes that the magnitude of a “typical” stimulus is represented by an internal reference. This internal reference evolves throughout an experiment and is updated on every trial. Different recent models have in common that they describe how the internal reference is computed but are agnostic to the decision process itself. In this study, we develop a new model that incorporates the mechanisms of perceptual discrimination models into a diffusion model. The diffusion model focuses on the dynamics of the decision process itself and accounts for choice and response times based on a set of latent cognitive variables. We show that our model accurately predicts the accuracy and response time distribution in a classical duration discrimination task. Further, model parameters were sensitive to the Type A and B effect. The proposed model opens up new opportunities for studying human discrimination performance (e.g., individual differences).

## Introduction

**Fig. 1:**
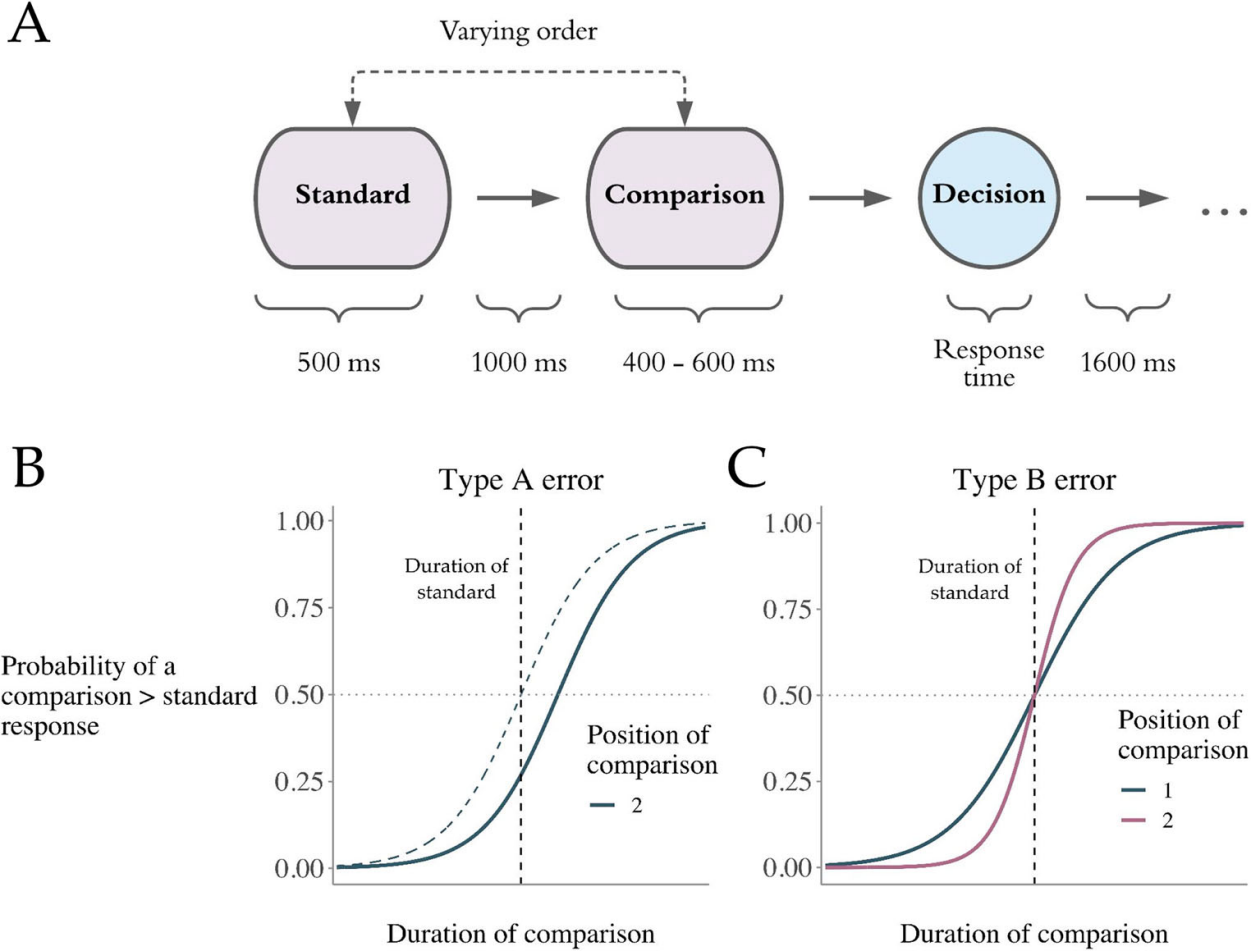
**A.** Timeline of the experimental duration discrimination task used in Dyjas, Bausenhart, and Ulrich (2012). On each trial, a standard stimulus with a constant duration of 500 ms and a varying comparison stimulus with durations ranging from 400 ms and 600 ms were subsequently presented. The presentation order of the stimuli pseudo-randomly vary across trials. After the second stimulus was presented, the participants had to decide which of the stimuli had a longer duration. The response time was measured after the second stimulus was presented until a response key was pressed. **B.** Graph showing two theoretical psychometric functions that map the response probability to different durations of the comparison stimulus. The point where these curves cross the horizontal dashed grey line indicates the point of subjective equality. When the duration of the comparison is not equal the duration of the standard at this point then we observe a Type A error, which is the case with the blue solid curve. **C.** Graph showing response probabilities as a function of the duration of the comparison for the two presentation orders separately. The two psychometric functions differ in their slope and thus indicate that the discrimination performance depends on the presentation order (Type B error)

Comparative decisions are fundamental in humans’ everyday lives and have been extensively studied since the advent of psychophysics (Fechner, 1860; Hegelmaier, 1853). In a typical experiment, participants have to select one of two stimuli based on the magnitude of a specific stimulus feature. For instance, deciding which of two subsequently presented tones had a longer duration (see, e.g., Fig. 1A). A class of psychophysical models, called *difference models* (Thurstone, 1927a, b), assumes that participants compare their internal representation of the two presented stimuli and base their decision on the difference in magnitude between these internal representations, *D* = *X*_1_ − *X*_2_.

Usually, this difference in stimulus magnitude (e.g., duration of tones) is experimentally manipulated by varying the intensity of one stimuli between trials. What is expected is that the difficulty of the decision depends on the difference between the two stimulus intensities. Deciding between stimuli with a relative large difference in intensity is easier than compared to stimuli that are very similar. Participants’ performance in such tasks can be described with a (*sigmoidal*) psychometric function that maps varying stimulus intensities to the proportion of a certain response. The steepness of the slope of this psychometric function indicates the individual’s sensitivity to differences in stimulus magnitude (see, e.g., Fig. 1B).

Many studies have shown that discrimination performance (often indexed by the *difference limen*; *DL*^1^) in such tasks is not only influenced by the stimulus intensities but also by task-irrelevant features of the experimental context (for a recent review see, Bausenhart et al., 2016) – effects that classical difference models cannot explain. Therefore, more complex models have been proposed with the aim of accounting for such context effects. However, while these models often accurately describe the discrimination performance they are agnostic about the decision process itself. In the present study, we present a new approach that aims to incorporate variants of the state of the art model of perceptual discrimination into a decision process model. Although studies have investigated perceptual discrimination and the influence of contextual effects in a wide variety of stimulus features, the focus of the present study lies on the discrimination of short stimulus durations (below 1 s). In what follows, we briefly explain typical context effects in discrimination tasks and how these are accounted for, and then describe our modeling approach.

As noted above, decisions based on subjective stimulus intensity estimates can be biased by various contextual factors (so-called *carryover effects*). These effects can be broadly divided into *perceptual* and *decisional* context effects. Whereas the former refers to biases that occur based on the perception of previously encountered stimuli, the latter describes contextual effects as a result of prior decisions. Every context effect can either be *assimilative* (i.e., the perception or decision is pulled towards previous ones) or *contrastive* (i.e., the perception or decision diverges from previous trials; Wiener et al., 2014). Further, these effects can broadly be classified into *global* or *local* effects. Global context effects describe the impact of the total set of past stimuli or decisions on a given trial. Conversely, *local* effects refer to the influence of immediately preceding trials (de Jong, Akyürek, & van Rijn, 2021). A typical example of a perceptual context effect is the central-tendency effect, also known as Vierordt’s law (Vierordt, 1868; Lejeune & Wearden, 2009). According to this law, humans tend to overestimate relative short durations and underestimate relative long durations (e.g., see Karin M. Bausenhart, Dyjas, & Ulrich, 2014; Grondin, 2005; Gu & Meck, 2011; Taatgen & van Rijn, 2011). Humans, thus, show a regression to the mean where the single stimulus is biased towards a representation of an average of previous stimuli (this reflects an assimilative global effect).

The so-called *Type A effect*, also known as the *time-order error* (TOE), refers to the impact of stimulus order on the *point of subjective equality* (PSE; Fechner, 1860). This means that participants usually over- or underestimate one stimulus relative to the other depending on the presentation order (for a comprehensive review of this research see, Hellström, 1985). Figure 1B shows a graph with two psychometric functions. The blue dashed sigmoid curve shows equal choice probability of the two responses (comparison > standard; comparison < standard) when the comparison and standard stimulus had the same duration. This means that there was no systematic over- or underestimation of either stimuli. The solid blue line has the same slope but is horizontally shifted to the right. This shows us that the duration of the standard stimulus frequently overestimated, which means there is a Type A effect.

In the classical *difference model* the Type A effect is accounted for by assuming a response bias parameter as an additive constant (Yeshurun, Carrasco, & Maloney, 2008; Alcalá-Quintana & Garcáa-Pérez, 2011). However, subsequent studies have invalidated this assumption at least to some extent (Hellström, 1977; Jamieson & Petrusic, 1976). These studies suggest that the cause of the TOE lies in perceptual – that is, pre-decisional – processes, which again cannot be explained by the *difference model*. However, possible decisional biases cannot be ruled out entirely. To date, the interplay of perceptual decisional processes in the origin of the TOE are not fully understood.

Dyjas et al., (2012) showed that when participants have to discriminate the duration between a constant standard stimulus *s* and a varying comparison *c* their discrimination performance is better when the standard stimulus precedes, rather than follows, the comparison stimulus. This effect is often referred to as a negative Type B effect^2^ (Ulrich & Vorberg, 2009) and has also been shown in other domains such as *weight* (Ross & Gregory, 1964) or *contrast* discrimination (Nachmias, 2006). The Type B effect reflects a decreased slope of the sigmoid function for trials with the stimulus order [cs] compared to trials with reversed order (see, Fig. 1C). The Type A effect, however, reflects merely a lateral shift of the sigmoid function mapping response probabilities to the difference in stimulus duration. The Type B effect – albeit being observed across different modalities (e.g., visual, auditory) and stimulus attributes (e.g., duration, frequency, intensity, and numerosity) – received much less attention in research (Ellinghaus, Gick, Ulrich, & Bausenhart, 2018). Although most studies found a negative Type B effect (i.e., better discrimination when the comparison stimulus is presented after the standard), some studies found a reversed effect (Hellström, Patching, & Rammsayer, 2020), especially, when stimulus duration and the inter-stimulus-interval (ISI) are very short (≤ 300 ms).

Both Type A and Type B effects are assumed to be *global* context effects, in the sense that their cause lies in the history of many previously encountered stimuli. Raviv, Ahissar, and Loewenstein (2012) demonstrated with an absolute stimulus duration identification task that immediately preceding trials are positively correlated. To investigate whether this effect is due to the perception of the previous stimulus or due to the previous decision, Wiener, Thompson, and Coslett (2014) conducted a study in which they counterbalanced the order of the different stimuli. They observed that decisions biased perception in the following trial, such that the interval was judged similarly. Further, they also found a *contrast* effect of stimulus perception on the subsequent perception. Further, an order effect on PSE (Type A effect) has also been observed (Dyjas, Bausenhart, & Ulrich, 2012; de Jong et al., 2021). Altogether, several *global* and *local* effects influence perception and decisions in discrimination tasks which cannot be explained by merely considering differences in stimulus magnitudes.

The predominant explanation for all these context effects is that decisions concerning the magnitude of a stimulus feature (e.g., duration) are derived not only based on the currently presented stimulus but also on the distribution of previously encountered stimuli. Thus, it is apparent that information stored in the memory system influences perception of and decisions about later presented stimuli. More recent modeling approaches aiming to improve the theoretical accounts for context effects on duration discrimination performance all share the theoretical rationale that responses to interval timing are based on a triad of cognitive processes: (1) A perceptive clock system that systematically changes over time. (2) A temporal reference memory system that stores past encounters with the stimulus. And, (3) a decision process that determines how the current output of the perceptive system relates to the values stored in the memory system and how to take any action based on this comparison (for a recent review see, van Rijn, 2016). Many different models have been proposed, which try to explain how the brain keeps track of time and implements such a clock system for time perception (for a review see, Balcı & Simen, 2016).

### Internal reference model

The reference memory system in particular is assumed to play an important role in the occurrence of context effects. Lapid, Ulrich, and Rammsayer (2008), for example, assume that participants store an internal reference of a prototype stimulus in the memory and update this reference over time (e.g., Durlach & Braida, 1969). Dyjas et al., (2012) proposed a quantitative model, the *internal reference model* (IRM), that describes how such an internal reference (*I*) is established and updated over time. According to this model, the internal reference *I*_*n*_ on a given trial *n* is computed as a weighted sum of the internal reference of the previous trial *I*_*n*− 1_ and the internal representation *X*_1,*n*_ of the first stimulus of the actual trial. This means that the internal reference is updated on a trial-by-trial basis, such that the internal reference *I*_*n*_ on trial *n* follows a geometrically moving average (Roberts, 1959):

1 $$ I_{n} = g \cdot I_{n-1} + (1 -g) \cdot X_{1,n}, $$

with a weight *g*, 0 ≤ *g* ≤ 1. This parameter indicates how much weight is given to the internal reference. To make a decision, participants compare this internal reference *I*_*n*_ with the internal representation of the second stimulus *X*_2,*n*_, *D*_*n*_ = *I*_*n*_ − *X*_2,*n*_. When this difference *D*_*n*_ is greater (smaller) than 0 then they decide that the first stimulus was longer (shorter). This model simplifies to the standard difference model if the weight *g* is set to 0.

Different studies showed that the IRM succeeds in predicting various context effects such as Vierort’s law (Bausenhart, Dyjas, & Ulrich, 2014), (Bausenhart et al., 2014), Type A and B effect, as well as n-1 effects (Dyjas et al., 2012; Dyjas & Ulrich, 2014; Bausenhart, Dyjas, & Ulrich, 2015; Ellinghaus, Ulrich, & Bausenhart, 2018), comparison stimulus precedes a constant standard stimulus, the internal reference is no longer stable across trial because the variable stimulus gets integrated. This variation of the internal reference representation then causes a decreased discrimination performance. Thus, the size of the Type B effect, for example, should increase with increasing *g* because the percept is then influenced more strongly by the varying internal reference (Dyjas & Ulrich, 2014). A recent study by Ellinghaus et al., (2018) showed that this weight decreases when the interval between two stimuli increases. The idea behind this finding is that the internal representation decays over time. Dyjas et al., (2012) showed in two experiments that this model successfully accounts for the behavioral patterns (e.g., Type B effect) in a duration discrimination task where the stimulus order of a constant standard and a variable comparison stimulus was manipulated.

### Sensation weighting model

The *sensation weighting model* (SWM) proposed by Hellström (Hellström, 1979; 1985) is a more general account. This model does not incorporate a trial-by-trial updating of an internal reference and it is formalized as follows:

2 $$ D = [w_{1}X_{1} + (1-w_{1})R_{1}] - [w_{2}X_{2} + (1-w_{2})R_{2}] + b, $$

where *D* is the subjective difference between sensation magnitudes of two stimuli *X*_1_ and *X*_2_ each weighted by *w*_1_ and *w*_2_. The parameter *b* reflects a bias. *R*_1_ and *R*_2_ are reference levels (similar to the internal reference) that indicate the average subjective level of stimulation of the stimuli. The crucial difference of the SWM (compared to the IRM) is that not only the first but also the second stimulus has a corresponding internal reference. This model simplifies to the same discrimination process as the IRM with *s*_2_ = 1 and *b* = 0. Within this model context effects are explained by different weights for the stimuli. As shown by Hellström (1979) a larger weight for the second stimulus results in a Type B effect. Hellström, Patching, and Rammsayer (2020) argue that the SWM but not the IRM accounts for the full range of observed Type B and Type A effects. As soon as the standard stimulus is not fixed anymore (*roving standard tasks*), the IRM has problems accounting for the effects. Also, the sometimes observed positive Type B effects are difficult to explain with the IRM model (Hellström et al., 2020; de Jong et al., 2021). However, as Dyjas and Ulrich (2014) stated, it could be a promising approach to combine the generality of the SWM and the trial-by-trial updating mechanism of the IRM.

Both models discussed here ground on the notion of stimulus comparison, described by a linear model with different weights for the two stimuli and/or an integration of past stimulus experiences. Altogether, these models pose important progress compared to the standard difference model in accounting for various context effects. However, they focus on the memory system of the cognitive triad and are rather agnostic about the subsequent decision-making processes. The present study aims to provide a more detailed description of the processes involved in duration discrimination by incorporating the concepts of the IRM and the SWM into a *diffusion decision model* (DDM). In such a framework, choice and response time data are jointly analyzed. This allows for a more fine-grained analysis of the ongoing cognitive processes. It is well known that accuracy trades off with speed (e.g., Heitz, 2014). Accounting for response times can prevent potential inferential biases. Furthermore, using an additional source of information (response times) can act as a useful constraint for parameter estimation and can increase parameter recoverability (Shahar et al., 2019; Ballard & McClure, 2019).

### Diffusion decision model

The DDM, originally developed by Roger Ratcliff (Ratcliff, 1978; for recent reviews see, Ratcliff, Smith, Brown, & McKoon, 2016; Voss, Nagler, & Lerche, 2013), belongs to the broader class of *evidence accumulation models*, sometimes also referred to as *sequential sampling models*. Its core assumption is that noisy evidence is accumulated over time until a decision boundary (one for each decision alternative) is reached. This terminates the evidence sampling process, and the decision corresponding to the crossed boundary is made.

The standard DDM consists of four parameters, which all correspond to a specific cognitive aspect of the decision process: The drift rate *v* refers to the average rate of evidence accumulation. The boundary separation *α* is the distance between the boundaries and indicates how much evidence one considers necessary to reach a decision. Hence, this parameter is interpreted as a measure for response caution. The starting point *z* determines where the evidence integration process starts relative to the distance to the decision boundaries. If the starting point is equidistant from both decision boundaries, both decision alternatives have the same probability before the evidence accumulation process starts. When the starting point is shifted toward one of the boundaries, participants show an *a priori* bias toward one of the alternatives. Finally, the non-decision time *τ*, which is associated with the process of stimulus encoding and the execution of some action after one of the boundaries has been reached. The DDM is one of the most influential cognitive process models and is used in a wide variety of research domains involving two-alternative forced choice tasks (for a review see ; Wagenmakers, 2009). One of its advantages lies in the joint analysis of choices and the full response time distributions for correct and error responses. This allows the model, for example, to account for the prominent speed-accuracy trade-off (Luce, 1986; Heitz, 2014). Combining variants of the psychophysical memory models described above and the DDM into one framework could be beneficial for the field of stimulus discrimination and for the field of decision-making. On the one hand, it may be a fruitful extension for the models of perceptual discrimination, which are agnostic about the decision process itself and – on the other hand – it advances decision process models that usually ignore sequential and contextual effects in experiments. To account for the context effects (e.g., Type B effects) some of the DDM parameters must vary systematically from trial to trial. The DDM has already been extended by trial-by-trial variability parameters for the drift rate, starting point and also the non-decision time, which is often referred to the “full” Ratcliff diffusion model (Ratcliff & Tuerlinckx, 2002). It has been argued that these variabilities improve the quality of data fit, especially for fast responses (Lerche & Voss, 2016; Boehm et al., 2018). However, these trial-by-trial variabilities are usually assumed to be random. For the present model, our goal is to inform trial-by-trial variability, especially of the drift rate and the starting point, based on the proposed mechanics of the discrimination models described above.

To our knowledge, the study from Patching, Englund, and Hellström (2012) is the only attempt in this direction. In their study, the authors modeled data from a paired visual stimuli size and brightness discrimination experiment with a DDM that regressed the drift rate on differently weighted magnitudes of the stimuli:

3 $$ v_{n} = w_{1}X_{1,n} - w_{2}X_{2,n} + b, $$

where *X*_1,*n*_ and *X*_2,*n*_ are the stimulus magnitudes on a given trial *n* with their respective weights *w*_1_ and *w*_2_, and *b* is a constant. This corresponds to the mechanisms proposed by the SWM. Besides, they included also a random trial-by-trial variance for the drift rates, starting points and non-decision times. Their model succeeded in predicting the Type A effect. In the present study, we build on this study and apply different models with a similar rationale to empirical data from a duration discrimination experiment. First, we use these models to predict not only possible Type A effects but also the Type B effects. Second, we integrate different variants of the IRM and the SWM into a DDM framework and compare their fits to the data.

In discrimination task experiments, such as described earlier, response times (RT) are usually measured from the offset of the second stimulus. This means that the evidence accumulation process of the DDM technically starts when the presentation of the second stimulus ends. Balcı and Simen (2014) proposed a two-stage sequential diffusion model with a similar idea in mind. The first stage is a diffusion process that delivers a noisy estimate of a time interval, which arises from a balance between excitation and inhibition and is referred to as a *time-adaptive, opponent Poisson drift diffusion model* (TOPDDM). The starting point as well as the drift rate of the second diffusion process, which corresponds to the actual decision process required in the experiment, is then influenced by the first stage’s first passage time. Within the TOPDDM framework, different intervals are timed by adjusting the accumulation rate; a higher drift rate is used to time shorter intervals. It is worth mentioning that this sequential DDM was applied to experimental data from a bisection task. On each trial in this task, a single stimulus had to be classified in one of two categories. Therefore, the TOPDDM formalizes the perception and decision involving a single stimulus and cannot account for comparative decisions of two stimuli. Also, the model does not incorporate any memory system mechanism that could account for context effects. Still, the study showed that the perception of stimulus can influence the starting point of the subsequent evidence accumulation process.

We assume that information from the first stimulus additionally affects the starting point of the evidence accumulation. To evaluate whether our model assumptions are plausible, we fitted different models to the data from Dyjas et al., (2012). The relative fit to the data for all models was assessed with an approximate leave-one-out cross-validation procedure (Vehtari, Gelman, & Gabry, 2017). In addition to the relative goodness-of-fit it is important to also assess the absolute fit to the data (i.e., the degree to which they can capture quantitative and qualitative patterns in the empirical data), because even the relative best-fitting model could be a bad model for describing the data generating process (Palminteri, Wyart, & Koechlin, 2017). Therefore, we performed posterior predictive checks with the relative best-fitting model for response and response time data.

## Methods

This study is based on a re-analysis of the data form Dyjas et al., (2012). More details about the methods can be found in the original article.

### Participants

26 volunteers with normal hearing and sight participated in 3 sessions on different days in the first experiment. Data from 5 participants were eliminated from all analyses due to non-cooperative participation (see the *contaminants handling* subsection for more information about our elimination procedure). This resulted in a final sample *N* = 21 participants (15 female; 6 male) with an average age of 24.85 years (*SD* = 7.3, range = 18-41). For the second experiment, a novel sample consisting of 24 female participants was recruited. We excluded 3 individuals due to non-cooperative participation which resulted in a final sample of 21 participants with an average age of 20.19 years (*SD* = 2.6, range = 18-28).

### Experimental task

In Experiment 1, participants had to decide which of two subsequently presented auditory stimuli (white noise) had a longer duration. On each trial, a stimulus (*s*) had a standard duration of 500 ms, while for the comparison stimulus *c* durations ranging from 400 to 600 ms were used. The inter-stimulus interval was always 1000 ms. Participants had to decide whether the first or the second stimulus had a longer duration. Response times were recorded starting from the offset of the second stimulus until a response has been made. After an inter-trial interval of 1600 ms, the next trial began. The experiment consisted of three conditions that differed in the order of the two stimuli and were tested in separate sessions. In the [*sc*] *blocked* condition the standard stimulus *s* always preceded the comparison stimulus *c*. In the [*cs*] *blocked* condition, this order was reversed. In the *random* condition, both stimulus orders were presented randomly intermixed.

In Experiment 2, the task was the same except that visual (*discs*) instead of auditory stimuli were used and the range of durations of the comparison stimulus was increased to 300-700 ms. In the original study (Dyjas et al., 2012), no substantial differences have been found between the fixed and random conditions. For brevity, we focus our data analysis on the *random* condition of both experiments.

### Contaminants handling

Generally, it is important to have an appropriate strategy for handling data points that are not a product of the process in consideration but from another process that is not in the focus of the research question (*contaminants*; Zeigenfuse and Lee, 2010). *Fast guesses* are one type of such contaminants, which are very fast responses (e.g., ≤ 300 ms) with chance level performance. In the case of diffusion modeling, it is particularly important to appropriately deal with this type of contaminants because otherwise, it can lead to biased parameter estimation and incorrect standard errors (Ratcliff, 1993; Ratcliff & Tuerlinckx, 2002; Ulrich & Miller, 1994). Furthermore, an analysis of contaminants helps to detect non-cooperative participants who can then be excluded from further data analysis.

Therefore, we applied a method called *exponentially weighted moving average* (EWMA; Chandra, 2007; Vandekerckhove & Tuerlinckx 2001) to identify fast guesses for each individual separately. If the proportion of fast guesses exceeded 10% of all responses then this participant was excluded from further analyses. Some participants showed only a few very fast responses. In this case, it is not appropriate to calculate average accuracy because performance could exceed chance level randomly. Therefore, we additionally removed all responses faster than 100 ms.

### Cognitive process models

With this study, we want to evaluate whether the DDM is an appropriate model for explaining decision processes involved in duration discrimination. Our core assumption is that – when two stimuli are presented sequentially – then the first stimulus influences the starting point of the evidence accumulation process, while the drift rate depends on the magnitude of both stimuli. We further enrich the diffusion model with a memory model that determines the influence of previously seen stimuli, as proposed by different variant of the IRM, SWM or a combination of both. We estimated several different models to test whether these assumptions are justified given our data (see Table 1 for an overview of all tested models).

**Table 1:** The different specifications of DDMs with the number of free individual-level parameters, their goodness of relative fit to the data quantified by the expected log-predictive density (elpd), and the corresponding uncertainty of these values quantified by the standard error (SE)

Model	Specification	N pars	Exp 1		Exp 2	
			elpd	*SE*	elpd	*SE*
1	*v*_*n*_ = *v*_0_ + *v*_1_(*X*_1,*n*_ − *X*_2,*n*_)	5	− 6925	148	− 4767	121
2	*v*_*n*_ = *v*_0_ + *v*_1_(*I*_1,*n*_ − *X*_2,*n*_)	6	− 6709	148	− 4636	121
3	*v*_*n*_ = *v*_0_ + *v*_1_*X*_1,*n*_ + *v*_2_*X*_2,*n*_	6	− 6706	148	− 4630	121
4	*v*_*n*_ = *v*_0_ + *v*_1_*I*_1,*n*_ + *v*_2_*X*_2,*n*_	7	− 6681	148	− 4609	121
5	*v*_*n*_ = *v*_0_ + *v*_1_(*I*_1,*n*_ − *X*_2,*n*_)					
	*z*_*n*_ = *z*_0_ + *z*_1_*I*_1,*n*_	7	− 6677	148	− 4597	120
6	*v*_*n*_ = *v*_0_ + *v*_1_*X*_1,*n*_ + *v*_2_*X*_2,*n*_					
	*z*_*n*_ = *z*_0_ + *z*_1_*X*_1,*n*_	7	− 6637	147	− 4591	120
7	*v*_*n*_ = *v*_0_ + *v*_1_*I*_1,*n*_ + *v*_2_*X*_2,*n*_					
	*z*_*n*_ = *z*_0_ + *z*_1_*I*_1,*n*_	8	− 6611	147	− 4609	121

*v*_*n*_ and *z*_*n*_ refer to the drift rate and starting point, respectively, on a given trial *n*. *X*_1,*n*_ and *X*_2,*n*_ denote the internal representation of the first and second stimulus on trial *n*. *I*_1,*n*_ is the internal references computed by the mechanism suggested by the IRM (Equation 1)

Model 1 is a baseline model, which incorporates the simple difference model. Here, the drift rate is the only parameter that is allowed to vary between trials. This is modeled as a linear function of the difference between stimulus durations on the present trial. This model comprises a total of 5 free individual-level parameters: The intercept and slope of the drift rate, the starting point, the boundary separation, and the non-decision time.

Models 2-4 differ from the Model 1 in the linear function describing the drift rate. Model 2 implements the concept of the IRM by replacing the internal representation of the first stimulus *X*_1_ with the internal reference *I*_1_, which is calculated for every trial following Equation 1. This introduces one additional parameter *g* that weights past internal references and the currently present first stimulus. In Model 3, the drift rate is calculated according to the SWM. Here, the drift rate *v* on a given trial *n* is modeled as a linear function of an intercept *v*_0_ and different weights (*β*_1_, *β*_2_) for the first and the second stimulus. Model 4 implements a combination of the IRM and SWM concepts, as suggested by Dyjas and Ulrich (2014). This model uses not only an internal reference for the first stimulus but also allows for different weighting of both presented stimuli.

To examine whether the processing of the first stimulus influences the starting point of the evidence accumulation process we refitted the Models 2 to 4 with an additional linear function for the starting point *z* (Models 5-7). For simplicity reasons, we did not include the random trial-by-trial variability parameters like Patching et al., (2012) did. We fitted all 7 models to the data of the *random* condition of Experiment 1 and 2.

### Model fitting and evaluation

All models were implemented in a Bayesian hierarchical framework (Vandekerckhove, Tuerlinckx, & Lee, 2011). For each parameter, a Gaussian hyper-distribution was estimated from which individual parameters for each participant were sampled. This procedure allows to investigate inter-individual differences in the stimulus comparison process and also serves to account for a source of variability in the average parameter estimates (Lee, 2011). See Appendix A for a description of all (hyper-) priors used in our models.

All models were implemented in Stan (Carpenter et al., 2017; Stan Development Team, 2020b) and estimated with the R interface package RStan (Stan Development Team, 2020a). Samples were drawn using a Hamiltonian Monte Carlo sampler (HMC; Betancourt, 2018) with 4 chains and 2000 iterations of which 50% were used as warm-up samples and later discarded. To ensure model convergence we inspected the $\hat {R}$ statistic (Gelman & Rubin, 1992) and assured that $\hat {R} < 1.01$ for all parameter estimates. We compared the relative fit of all models using *approximate leave-one-out cross-validation* (Loo R package; Vehtari et al., 2020; Vehtari et al., 2017). The best-fitting model, indicated by the highest expected log-predictive density (elpd), was then rigorously evaluated in terms of absolute fit by the means of posterior predictive checks. We sampled 500 parameters set from the posterior distribution. We then simulated new datasets with these parameters and calculated summary statistics for the responses (*mean* and 95% *highest density interval*; HDI) as well as response times (*median* and 95% HDI) and compared those with summary statistics of the empirical data. All code and data are freely available on GitHub (https://github.com/LuSchumacher/timing_discrimination).

### Individual context effects

In order to evaluate the degree of Type B and Type A, effect we fitted a Bayesian logistic regression to the data for each participant separately. This model predicted the proportion of a *c* > *s* response with the predictors *duration of* *c*, *stimulus order*, and their interaction. We then calculated the difference limen for both stimulus orders separately by the difference between the 75% and 25% percentile of the resulting psychometric function divided by 2. The individual Type B effect was then determined by the difference between those two *DL*’s. The individual Type A effect was calculated from the difference between the *PSE* of both stimulus orders, which corresponded to the 50% percentile of the psychometric function.

## Results

### Relative model fit

**Fig. 2:**
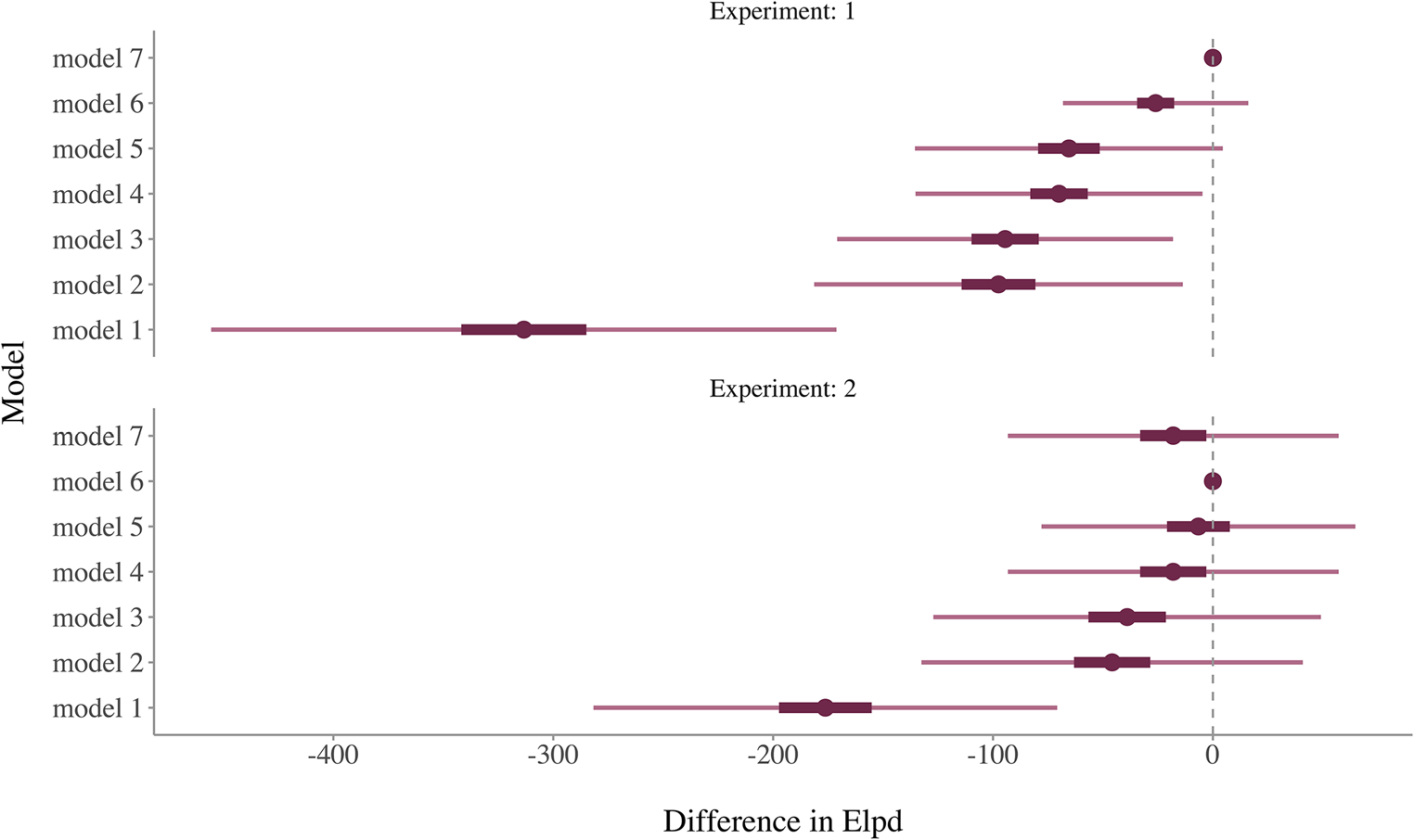
Differences in the expected log-predictive density (elpd) for each model fitted to the data of Experiments 1 and 2. The difference for each model is computed relative to the best-fitting model, and thus, the elpd difference of the best-fitting model equals 0. The thick bars indicate ± 1 standard error and the thin bar ± 5 *SE*’s of the elpd difference estimate

The differences in relative goodness-of-fit for all models are depicted in Fig. 2 for both experiments separately. All models’ elpd values were compared relative to the best-fitting model. Hence, the model with the most accurate out-of-sample prediction has an elpd difference of 0. We clearly see that Model 1, which is an implementation of the simple difference model, performs worse compared to all other models in both datasets. The elpd values for the Models 2 and 3 are superior compared to Model 1 and fairly similar to each other (see Table 1). This suggests that the different implementations of the IRM and SWM predict data equally well. Model 4 which used different weighting of the stimuli as well as the internal reference mechanism showed a slightly better fit in both experiments compared Model 2 and 3.

Models 5 to 7 included an additional linear function for the starting point of the evidence accumulation process. These models tend to show a slightly better goodness-of-fit. However, we did not observe a clear increase in prediction accuracy when compared to model variants that did not include this additional predictor (Models 2–4). Again, the results differ between Experiments 1 and 2. In Experiment 1, Model 7, which is a combination of the IRM and SWM, showed the best goodness-of-fit. It appeared to predict the data more accurately compared to the model that was identical but without the linear function of the starting point (Model 4). This was not the case in Experiment 2. Neither was Model 7 the best-fitting model nor did it differ from Model 4 in a meaningful way. Our data suggest a large degree of model mimicry and it appears that the impact of the first stimulus (or the internal reference) on the starting point is rather small.

### Absolute model fit

**Fig. 3:**
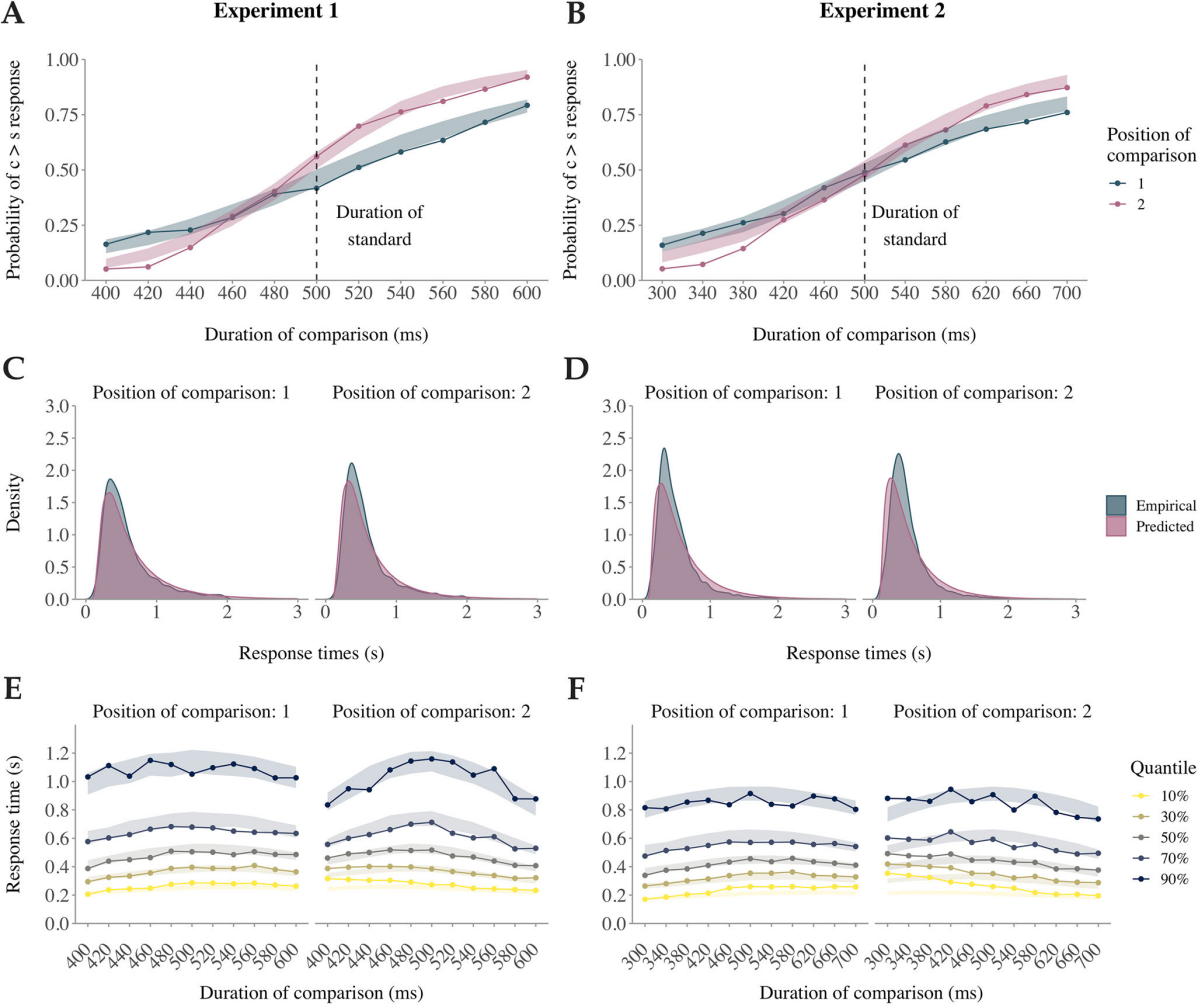
Posterior predictions and empirical data of the average performance in Experiment 1 and 2. **A**, **B**. The average probability of a *c* > *s* response as a function of the duration of the comparison stimulus for both stimulus orders separately. The posterior prediction is shown with shaded areas (95% HDI) and the empirical data with solid lines and points. **C**, **D**. The densities of the predicted and the empirical raw response time distribution of all participants for both stimulus orders separately. **E**, **F**. Different quantiles of the predicted (shaded area) and empiric (solid lines and points) average response time as a function of the duration of the comparison stimulus for both stimulus orders separately

Across both Experiments, Model 6 provided a good relative fit to the data. It predicted data best in Experiment 2 and was only slightly worse than the best-fitting model (Model 7) in Experiment 1 while using one less parameter. Thus, we evaluated this model in more detail in terms of absolute fit to data by performing posterior predictive checks. Figure 3AB depict the probability of deciding that the comparison stimulus *c* was longer than the standard stimulus *s* for all durations of *c* and both stimulus orders separately. The solid lines and points indicate the empirical data averaged across all participants. The shaded areas indicate the 95% HDI of the mode from 500 simulated datasets and thus, describe the model’s prediction of the average performance and its uncertainty. As expected, the probability of choosing *c* > *s* increases with increasing duration of c in both experiments. The slope of the line is steeper when the comparison followed rather than preceded the standard stimulus. This indicates a negative Type B effect. Both patterns were successfully predicted by the model. However, the model slightly overestimates the average probability of *c* > *s* responses for short durations of c in both experiments. This is probably due to shrinkage, which is a result of our hierarchical modeling approach. When the same model was fitted with complete pooling these divergences disappeared. Appendix A shows the posterior predictive checks based on the individual-level parameters for each participant separately. In these analyses, accurate predictions for all subjects were observed.

The empirical and predicted raw RT distributions are shown in Fig. 3CD for both stimulus orders and experiments separately. Comparing the empirical and the predicted densities reveal acceptable overlap in both conditions and experiments. However, the model was not able to capture the empirical data with very high precision. The mode of the predicted distribution was lower compared to the empirical distribution. Also, the model predicted heavier tails than have been observed in the data. All misfits were slightly more pronounced in Experiment 2 compared to Experiment 1 (see the Discussion section for possible explanations).

Figure 3EF shows a more fine grained picture of the RTs by depicting different quantiles (10%; 30%; 50%; 70%; 90%) of the observed and posterior predictive RT distributions for both stimulus orders and durations of stimulus *c* separately. In Experiment 1, RTs tend to increase with increasing task difficulty. This pattern seems to be more extreme in the tails of the RT distribution when stimulus *c* followed rather than preceded the standard stimulus. Both patterns were successfully predicted by our model. However, the uncertainty (95% HDI) increased in the tails of the RT distribution. This is due to the lower number of trials with such high RTs and also due to the greater variance.

The prediction of the RTs of Experiment 2 was not as good as for Experiment 1. Empirical RTs tend to be faster with short-duration *c* stimuli compared to longer durations if the comparison stimulus was presented first. The opposite pattern was found for the reversed order. A similar pattern was also observed in Experiment 1 although less pronounced. This is not a pattern we commonly would expect. Usually, RTs tend to increase with difficulty. Here, the most difficult decisions have to be made when the standard and comparison stimulus are the same or very similar. However, depending on the order of the stimuli either trials with short or long durations showed the slowest RTs. This is a pattern, which our model could not predict.

### Parameter specific analyses

Table 2 shows the mode and 95% HDI of the group-level mean parameter posterior distributions for both experiments separately. The posteriors of the boundary separation are similar between both experiments and show plausible values. The estimates for the non-decision time are again similar and lower than typically observed in cognitive experiments. This could be a result of the experimental paradigm. We discuss this issue in more detail in the *discussion* section. Remember, the starting point of this model was modeled as a linear function of the duration of the first stimulus. *z*_1_ corresponds to the beta-weight for the predictor *first stimulus* and its posterior is very small in both experiments. This means that in trials where the first stimulus showed the most extreme duration (-100 ms or 100 ms when centered on the standard stimulus) the influence of the first stimulus on the starting point would still be small (e.g., 0.0007 ⋅ 100 = 0.07 units of change in the starting point). Although this effect is very small, it is not zero.

**Table 2:** The mode and lower/upper boundaries of the 95% HDI of all group-level mean posterior distributions for both experiments separately

Parameter	Experiment 1			Experiment 2		
	mode	lower	upper	mode	lower	upper
*a*	1.4736	1.3577	1.5986	1.3921	1.3320	1.4597
*ndt*	0.1456	0.0986	0.1760	0.1226	0.0889	0.1468
*z*_0_	0.4411	0.4250	0.4559	0.4192	0.3967	0.4402
*z*_1_	0.0007	0.0005	0.0009	0.0002	0.0001	0.0003
*v*_0_	0.0437	− 0.0769	0.1671	0.2327	0.0959	0.3480
*v*_1_	0.0096	0.0073	0.0120	0.0060	0.0047	0.0075
*v*_2_	− 0.0198	− 0.0225	− 0.0170	− 0.0087	− 0.0095	− 0.0078

**Fig. 4:**
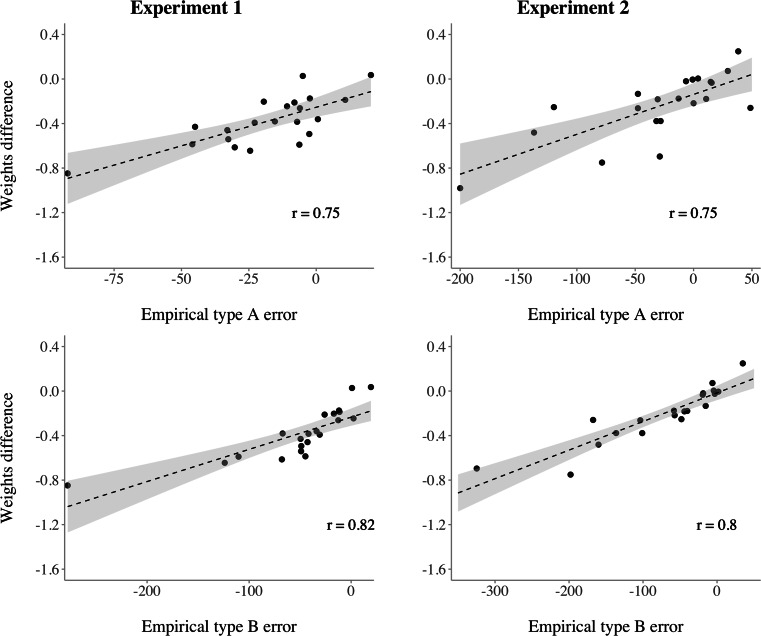
Correlation between the proportional difference in weights and the empirically observed Type A (**A**) and Type B effect (**B**) of Experiment 2 (left panel) and Experiment 2 (right panel). Each point corresponds to a single participant’s context effect and their individual-level proportional difference in weights

As pointed out by Dyjas and Ulrich (2014) and Hellström et al., (2020), the Type A and Type B effect are explained within the SWM as a result of the different weighting of both stimuli. Figure 4 shows the correlation between the individual proportional difference between the weighting parameters ($\frac {v_{1} - v_{2}}{v_{1} + v_{2}}$) and the empirical Type A and Type B effect in Experiment 1 (left panel) and Experiment 2 (right panel). All participants except one showed either a tendency or a clear negative Type B effect which was already reported in the original study (Dyjas et al., 2012). We observe a large correlation of *r* = 0.82, which suggests that individual estimates of the weighting parameters are meaningful predictors for the size of individual Type B effects. Although a smaller association between the weights and the empirical Type A effect was found, a similar conclusion can be drawn.

## Discussion

Human performance in duration discrimination is influenced by several context effects. For instance, the order of two successively presented stimuli not only affects the point of subjective equality (Type A effect) but also the discrimination sensitivity (Type B effect). Current models that account for such effects propose that an internal representation of the stimulus history interferes with the perception of the current stimulus. Although they describe how a decision variable evolves, they are agnostic to the dynamics of the decision process itself.

In this work, we presented a novel modeling approach for perceptual decision-making in duration discrimination. We demonstrate that integration of current models of stimulus discrimination (IRM, SWM) into a Bayesian hierarchical diffusion decision model offers good prediction of the average as well as individual discrimination performance by taking not only responses but also the entire response time distribution into account. Moreover, we demonstrated that the estimates of the model parameters are meaningful predictors for two intensively studied context effects, the Type B effect and the Type A effect.

In the field of perceptual stimulus comparison two different models have been proposed: the internal reference model, and the sensation weighting model. To this date, it remains unclear, which of those models best describes human discrimination processes. Little effort has been made, to rigorously compare the prediction of these models. The present study is a step towards this direction. However, we observed large model mimicry and were not able to discriminate between the different models based on the empirical data. This is not too surprising because the authors of the original study pointed out that it makes little difference whether the IRM or SWM is applied (Dyjas et al., 2012). We think, it would be a promising avenue for future investigation to compare the different models with our framework based on more complex task such as, for example, the roving standard task or task with very brief stimulus presentations. Furthermore, these models could also be tested against various local context effects (Wiener, Thompson, & Coslett, 2014; de Jong et al., 2021). Our approach could prove particularly useful since effects on response times are often found in such experiments (Wiener et al., 2014).

Our hierarchical modeling approach showed accurate prediction of the performance of most individual participants. Individual weight parameters *w*_1_ and *w*_2_ for the SWM were estimated. We showed that these parameters highly correlated with the individual extent of the Type A and Type B effect. It has already been pointed out that individuals can significantly differ in context effects (Dyjas, Bausenhart, & Ulrich, 2014). The ability to estimate individual parameters that correspond to these effects combined with individual latent variables provided by the DDM could be fruitful for future studies on individual differences in stimulus discrimination.

Future work also should address the whole range of sequential effects. Differentiation between global and local context effects and also the difference between perceptual and decisional carryover effects which could all be either assimilative or contrastive. Wiener et al., (2014) found that the perception of time is susceptible to similar adaptive and decisional effects as other categorical stimuli, where the responses for any given interval are simultaneously assimilated by the prior response and contrasted away from the prior interval. Urai, de Gee, Tsetsos, and Donner (2019), for example, showed that previous choices biased the average rate of evidence accumulation and not, as previously thought, the starting point of the accumulation process. It would be interesting to see whether this is also the case in duration discrimination tasks.

The models proposed in this study mathematically formalize two aspects of the cognitive triad involved in interval timing discrimination; an updating process of a memory system such as the internal reference in the IRM and a decision process formalized by the diffusion decision model as an evidence accumulation process. However, the model does not include a perception mechanism such as a clock system implemented. Several promising models such as the TOPDDM (Balcı & Simen, 2014) or the pace-maker accumulator model exist (Church, 1984; Gibbon, Church, & Meck, 1984; Hartcher-O’Brien, Brighouse, & Levitan, 2016). Implementing such a mechanism into the modeling framework proposed here could lead to an even more complete formalization of all the processes involved in interval timing discrimination and could provide a promising framework for future studies. Moreover, recent advances in joint modeling enable us to incorporate neural data into diffusion models (Ghaderi-Kangavari, Rad, & Nunez, 2022; Turner, Forstmann, & Steyvers, 2019), which could be a fruitful approach to studying brain-behavior relationships during duration discrimination.

Further, Toso, Fassihi, Paz, Pulecchi, and Diamond (2021) showed that non-temporal stimulus features (e.g., loudness of a tone) can influence the perceived stimulus duration. In their duration comparison task, participants’ responses were biased depending on the intensity of the stimulus which resulted in a horizontally shifted psychometric curve. They argue that this bias is a perceptual rather than a decisional phenomenon because it occurred whether the non-relevant feature was manipulated in the first or the second stimulus. In their opinion, the first stimulus is dissociated from any decisional process. The models proposed in the present study could precisely test this assumption as the starting point of the evidence accumulation process directly represents a decisional bias.

In perceptual decision-making tasks, RTs heavily depend on the difficulty of a given trial, expecting longer decision times for relatively difficult trials. In this study’s experiments, the difficulty of a trial was relatively high when the duration of the comparison and the standard stimulus was very similar. The task difficulty decreases with larger duration differences because it gets more obvious which of the two stimuli’ duration was longer. Thereby trials with relatively short durations of *c* and relatively long durations should be equally difficult. As the difficulty of a trial decreases, also the RTs should decrease and not differ between trials with short and long *c* durations.

Surprisingly, that is exactly what was observed in the empirical data. In both stimulus order conditions, RTs for relatively long durations of *c* differed from RTs when relative short durations were presented. When the *c* stimulus preceded the *s* stimulus participants responded slower when relative long durations were presented compared to relatively short durations. The opposite pattern was found when the *c* stimulus followed the *s* stimulus. In this case, participants tend to show slower RTs in trials when relative short durations of *c* were presented. Therefore, RTs were generally slower when the first stimulus was clearly longer than the second stimulus compared to trials when the first stimulus had a shorter duration than the second one.

This particular pattern in empirical RT data was most pronounced in Experiment 2 but also present in Experiment 1. Our model was not able to capture such RT differences. This is not surprising as the model assumes that the drift rate depends on the weighted difference between the duration of the stimuli presented in a trial. If this difference is low, the model produces more slow and erroneous responses. Conversely, when the weighted difference is relatively large, the model predicts more accurate and faster responses, independent of whether the duration of *c* was short or long.

It is an important open question if this pattern in the RTs can be explained as a phenomenon of the perceptual decision-making process in duration discrimination or whether it is a result of the experimental task used to measure the discrimination performance. In this study’s experiments, the RTs were measured from the offset of the second stimulus until a response button was pressed. A possible explanation for this asymmetry in RTs could be that in trials where the duration of the first stimulus is shorter, participants can already know their choice around the time when the duration of the second stimulus exceeds the duration of the first stimulus. This would lead then to relatively fast responses as participants are already waiting for pressing the response button before they are allowed. Conversely, in trials where the first stimulus was longer than the second one, participants could be surprised by the abrupt ending of the second stimulus. This could then lead to delayed start-ups and thus explain the longer RTs in those trials.

Further, it is worth mentioning that some posteriors of the individual-level non-decision time parameters were odd. The mode of these distributions sometimes took on unrealistic small values of below 100 ms (range: 0.019 to 0.302). It is rather implausible that it took a participant only 19 ms to execute the motor action that was needed to give a response. This also contributes to the assumption that participants with such low non-decision time parameters at least sometimes already decided and prepared their response before the presentation of the second stimulus has finished.

Although we took good care of potential fast guesses by applying the EWMA method to each participants’ responses, a substantial proportion of all responses were very fast. These fast responses, however, were clearly above chance performance. Although we observed such fast above chance performance responses, these did not occur exclusively in the [cs] order but also in the [sc] order. As we have not programmed nor experienced the stimuli in the experiment it is hard to come up with a satisfying explanation for these odd findings and simply disclose that the estimates of the non-decision time in our study have to be interpreted with caution.

We suggest that future studies take a deeper look into these surprising behavioral patterns. One way could be to start the RT measurement from the onset of the second stimulus. This possibly leads to even shorter RTs in trials where the first stimulus is clearly shorter than the second because participants would no longer have to wait to press the response button until the second stimulus presentation is finished.

In summary, the model proposed in this work provides a novel approach to predict human performance in duration discrimination. It not only incorporates perceptual mechanism like stimulus weighting or internal representation updating but also decisional processes such as processing speed or decision caution. We think the proposed model lay a good starting point to further investigate perceptual and decisional context effects.

## Open Practices Statement

All data and code used in this study are freely available on GitHub (https://github.com/LuSchumacher/timing_discrimination). None of the experiments was preregistered.

## Figures and Tables

**Fig. 5:**
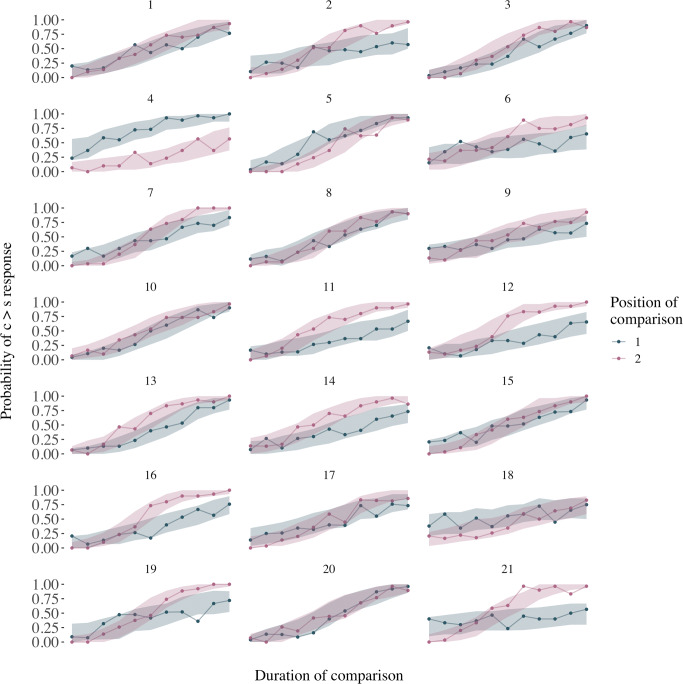
Posterior predictions (shaded area) and empirical data of Experiment 1 (solid line and points) of the mean probability of a *c* > *s* response for both positions of c and each participant separately. The shaded area represents the 95% HDI over the mean of 500 simulated datasets based on individual-level parameter sets randomly sampled from the posterior distribution

**Fig. 6:**
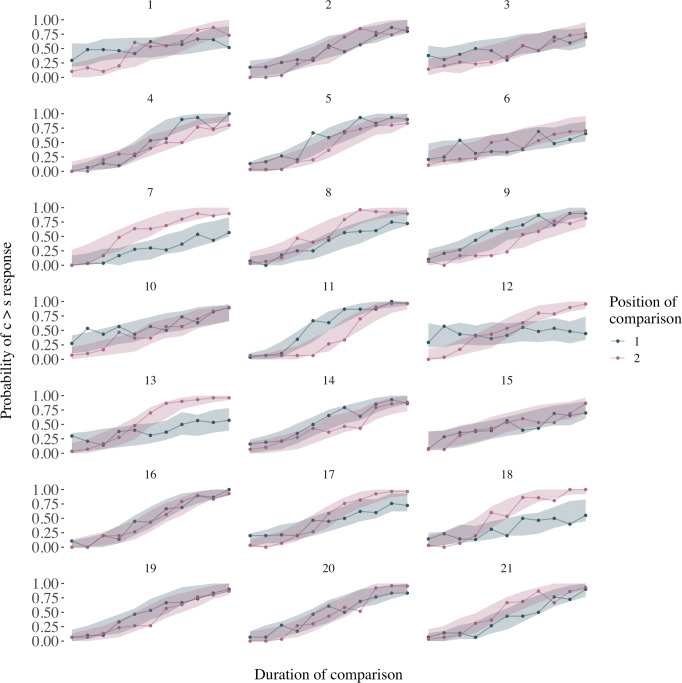
Posterior predictions (shaded area) and empirical data of Experiment 2 (solid line and points) of the mean probability of a *c* > *s* response for both positions of c and each participant separately. The shaded area represents the 95% HDI over the mean of 500 simulated datasets based on individual-level parameter sets randomly sampled from the posterior distribution

## Data Availability

All data used in this work are freely available on GitHub (https://github.com/LuSchumacher/timing_discrimination).

## Appendix A: Prior distributions

A list of the prior distributions used for all models. $\mathcal {N}$ refers to a Gaussian normal distribution with a mean *μ* and standard deviation *σ* parameter. Γ denotes a gamma distribution with a shape *κ* and scale *𝜃* parameter. The beta distribution is written as ${\mathscr{B}}e$ an uses two shape parameters *α* and *β*. All individual-level parameters were sampled from a group-level Gaussian normal distribution $\mathcal {N}(\mu ,\sigma )$, with a mean *μ* (listed below) and a standard deviation $\sigma \sim {\Gamma }(1,5)$. The group-level distributions for the boundary separation *α* and non-decision time *τ* parameter were truncated with a lower bound at 0. The group-level distribution for the *g* parameter, which governs the trial-by-trial updating mechanism of the internal reference was truncated with a lower bound at 0 and an upper bound at 1.

$$ \begin{array}{@{}rcl@{}} \mu_{v_{0}} &\sim \mathcal{N}(0,5)\\ \mu_{v_{1}}, \mu_{v_{2}} &\sim \mathcal{N}(0,1)\\ \mu_{z_{0}} &\sim \mathcal{B}e(5,5)\\ \mu_{z_{1}} &\sim \mathcal{N}(0,1)\\ \mu_{g} &\sim \mathcal{B}e(1,1)\\ \mu_{\tau} &\sim {\Gamma}(3,15)\\ \mu_{\alpha} &\sim {\Gamma}(2,2) \end{array} $$

## Footnotes

1 The *DL*, also called *just noticeable difference* (JND), is usually defined as the difference between the 75% and 25% percentile of a psychometric function divided by 2, $DL = \frac {x_{75} - x_{25}}{2}$, where *x* denotes the magnitude of a variable stimulus. This is a measure of the slope of the psychometric function where smaller values reflect better discrimination performance.

2 Rammsayer and Wittkowski (1990) called this effect the *position effect* and defined it the opposite way.

## Code Availability

The code used for the analysis in this study is freely available on GitHub (https://github.com/LuSchumacher/timing_discrimination).

## References

[CR1] Alcalá-Quintana R, Garcáa-Pérez MA (2011). A model for the time-order error in contrast discrimination. The Quarterly Journal of Experimental Psychology.

[CR2] Balcı F, Simen P (2014). Decision processes in temporal discrimination. Acta Psychologica.

[CR3] Balcı F, Simen P (2016). A decision model of timing. Current Opinion in Behavioral Sciences.

[CR4] Ballard IC, McClure SM (2019). Joint modeling of reaction times and choice improves parameter identifiability in reinforcement learning models. Journal of Neuroscience Methods.

[CR5] Bausenhart KM, Dyjas O, Ulrich R (2014). Temporal reproductions are influenced by an internal reference: Explaining the vierordt effect. Acta Psychologica, and Across Senses - Part-1.

[CR6] Bausenhart KM, Dyjas O, Ulrich R (2015). Effects of stimulus order on discrimination sensitivity for short and long durations. Attention, Perception, & Psychophysics.

[CR7] Bausenhart KM, Bratzke D, Ulrich R (2016). Formation and representation of temporal reference information. Current Opinion in Behavioral Sciences.

[CR8] Betancourt, M. (2018). A Conceptual introduction to Hamiltonian Monte Carlo. arXiv:1701.02434 [stat].

[CR9] Boehm U, Annis J, Frank MJ, Hawkins GE, Heathcote A, Kellen D, Krypotos A-M, Lerche V, Logan GD, Palmeri TJ, van Ravenzwaaij D, Servant M, Singmann H, Starns JJ, Voss A, Wiecki TV, Matzke D, Wagenmakers E-J (2018). Estimating across-trial variability parameters of the diffusion decision model: Expert advice and recommendations. Journal of Mathematical Psychology.

[CR10] Carpenter B, Gelman A, Hoffman MD, Lee D, Goodrich B, Betancourt M, Brubaker M, Guo J, Li P, Riddell A (2017). Stan: A probabilistic programming language. Journal of Statistical Software.

[CR11] Chandra MJ (2001). Statistical quality control.

[CR12] Church RM (1984). Properties of the internal clock. Annals of the New York Academy of Sciences.

[CR13] de Jong, J., Akyürek, E. G., & van Rijn, H. (2021). A common dynamic prior for time in duration discrimination. Psychonomic Bulletin & Review. 10.3758/s13423-021-01887-z 10.3758/s13423-021-01887-z PMC8367937 33661470

[CR14] Durlach NI, Braida LD (1969). Intensity perception: I. preliminary theory of intensity resolution. Journal of the Acoustical Society of America.

[CR15] Dyjas O, Bausenhart KM, Ulrich R (2012). Trial-by-trial updating of an internal reference in discrimination tasks: Evidence from effects of stimulus order and trial sequence. Attention, Perception & Psychophysics.

[CR16] Dyjas O, Bausenhart KM, Ulrich R (2014). Effects of stimulus order on duration discrimination sensitivity are under attentional control. Journal of Experimental Psychology: Human Perception and Performance.

[CR17] Dyjas O, Ulrich R (2014). Effects of stimulus order on discrimination processes in comparative and equality judgements: Data and models. Quarterly Journal of Experimental Psychology (2006).

[CR18] Ellinghaus, R., Gick, M., Ulrich, R., & Bausenhart, K.M. (2018). Decay of internal reference information in duration discrimination: Intertrial interval modulates the type B effect: Quarterly Journal of Experimental Psychology. 10.1177/1747021818808187 10.1177/1747021818808187 30282525

[CR19] Ellinghaus R, Ulrich R, Bausenhart KM (2018). Effects of stimulus order on comparative judgments across stimulus attributes and sensory modalities. Journal of Experimental Psychology. Human Perception and Performance.

[CR20] Fechner GT (1860). Elemente der psychophysik, Vol. 2.

[CR21] Gelman A, Rubin DB (1992). Inference from iterative simulation using multiple sequences. Statistical Science.

[CR22] Ghaderi-Kangavari, A., Rad, J.A., & Nunez, M.D. (2022). A general integrative neurocognitive modeling framework to jointly describe EEG and decision-making on single trials. 10.31234/osf.io/pqv2c

[CR23] Gibbon J, Church RM, Meck WH (1984). Scalar timing in memory. Annals of the New York Academy of Sciences.

[CR24] Grondin S (2005). Overloading temporal memory. Journal of Experimental Psychology: Human Perception and Performance.

[CR25] Gu, B.-M., & Meck, W.H. (2011). New perspectives on vierordt’s law: Memory-mixing in ordinal temporal comparison tasks. In A. Vatakis, A. Esposito, M. Giagkou, F. Cummins, & G. Papadelis (Eds.) *Multidisciplinary Aspects of Time and Time Perception: COST TD0904 International Workshop, Athens, Greece, October 7–8, 2010, Revised Selected Papers, in Computer Science* (pp. 67–78): Springer, DOI 10.1007/978-3-642-21478-3_6.

[CR26] Hartcher-O’Brien J, Brighouse C, Levitan CA (2016). A single mechanism account of duration and rate processing via the pacemaker-accumulator and beat frequency models. Current Opinion in Behavioral Sciences.

[CR27] Hegelmaier F (1853). Ueber Das Gedächtniss Für Linear-Anschauungen. Annalen der Physik.

[CR28] Heitz, R.P. (2014). The speed-accuracy tradeoff: History, physiology, methodology, and behavior. *Frontiers in Neuroscience*, 8. 10.3389/fnins.2014.00150 10.3389/fnins.2014.00150 PMC4052662 24966810

[CR29] Hellström Å (1977). Time errors are perceptual: An experimental investigation of duration and a quantitative successive-comparison model. Psychological Research Psychologische Forschung.

[CR30] Hellström Å (1979). Time errors and differential sensation weighting. Journal of Experimental Psychology. Human Perception and Performance.

[CR31] Hellström Å (1985). The time-order error and its relatives: Mirrors of cognitive processes in comparing. Psychological Bulletin.

[CR32] Hellström Å, Patching GR, Rammsayer TH (2020). Sensation weighting in duration discrimination: A univariate, multivariate, and varied-design study of presentation-order effects. Attention, Perception, & Psychophysics.

[CR33] Jamieson DG, Petrusic WM (1976). On a bias induced by the provision of feedback in psychophysical experiments. Acta Psychologica.

[CR34] Lapid E, Ulrich R, Rammsayer T (2008). On estimating the difference limen in duration discrimination tasks: A comparison of the 2AFC and the reminder task. Perception & Psychophysics.

[CR35] Lee MD (2011). How cognitive modeling can benefit from hierarchical bayesian models. Journal of Mathematical Psychology.

[CR36] Lejeune H, Wearden JH (2009). Vierordt’s the experimental study of the time sense (1868) and its legacy. European Journal of Cognitive Psychology.

[CR37] Lerche, V., & Voss, A. (2016). Model complexity in diffusion modeling: Benefits of making the model more parsimonious. *Frontiers in Psychology*, 7. 10.3389/fpsyg.2016.01324 10.3389/fpsyg.2016.01324 PMC5020081 27679585

[CR38] Luce RD (1986). Response times: Their rol in inferring elementary mental organization.

[CR39] Nachmias J (2006). The role of virtual standards in visual discrimination. Vision Research.

[CR40] Palminteri S, Wyart V, Koechlin E (2017). The importance of falsification in computational cognitive modeling. Trends in Cognitive Sciences.

[CR41] Patching GR, Englund MP, Hellström Å (2012). Time-and space-order effects in timed discrimination of brightness and size of paired visual stimuli. Journal of Experimental Psychology: Human Perception and Performance.

[CR42] Rammsayer T, Wittkowski KM (1990). Zeitfehler und positionseffekt des standardreizes bei der diskrimination kurzer zeitdauern. Zeitfehler und Positionseffekt des Standardreizes bei der Diskrimination Kurzer Zeitdauern.

[CR43] Ratcliff R (1978). A theory of memory retrieval. Psychological Review.

[CR44] Ratcliff R (1993). Methods for dealing with reaction time outliers. Psychological Bulletin.

[CR45] Ratcliff R, Smith PL, Brown SD, McKoon G (2016). Diffusion decision model: Current issues and history. Trends in Cognitive Sciences.

[CR46] Ratcliff R, Tuerlinckx F (2002). Estimating parameters of the diffusion model: Approaches to dealing with contaminant reaction times and parameter variability. Psychonomic Bulletin & Review.

[CR47] Raviv O, Ahissar M, Loewenstein Y (2012). How recent history affects perception: The normative approach and its heuristic approximation. PLOS Computational Biology.

[CR48] Roberts SW (1959). Control chart tests based on geometric moving averages. Technometrics.

[CR49] Ross HE, Gregory RL (1964). Is the weber fraction a function of physical or perceived input?. The Quarterly Journal of Experimental Psychology.

[CR50] Shahar N, Hauser TU, Moutoussis M, Moran R, Keramati M, Consortium N, Dolan RJ (2019). Improving the reliability of model-based decision-making estimates in the two-stage decision task with reaction-times and drift-diffusion modeling. PLOS Computational Biology.

[CR51] Stan Development Team (2020). RStan: The R interface to Stan. R package version 2.21.2. Retrieved from http://mc-stan.org/

[CR52] Stan Development Team (2020). Stan modeling language users guide and reference manual. https://mc-stan.org/.

[CR53] Taatgen N, van Rijn H (2011). Traces of times past: Representations of temporal intervals in memory. Memory & Cognition.

[CR54] Thurstone LL (1927). A law of comparative judgment. Psychological Review.

[CR55] Thurstone LL (1927). Psychophysical analysis. The American Journal of Psychology.

[CR56] Toso A, Fassihi A, Paz L, Pulecchi F, Diamond ME (2021). A sensory integration account for time perception. PLOS Computational Biology.

[CR57] Turner, B.M., Forstmann, B.U., & Steyvers, M. (2019). Joint models of neural and behavioral data. 10.1007/978-3-030-03688-1

[CR58] Ulrich R, Miller J (1994). Effects of truncation on reaction time analysis. Journal of Experimental Psychology: General.

[CR59] Ulrich R, Vorberg D (2009). Estimating the difference limen in 2afc tasks: Pitfalls and improved estimators. Attention, Perception, & Psychophysics.

[CR60] Urai AE, de Gee JW, Tsetsos K, Donner TH (2019). Choice history biases subsequent evidence accumulation. eLife.

[CR61] van Rijn H (2016). Accounting for memory mechanisms in interval timing: A review. Current Opinion in Behavioral Sciences.

[CR62] Vandekerckhove J, Tuerlinckx F (2007). Fitting the ratcliff diffusion model to experimental data. Psychonomic Bulletin & Review.

[CR63] Vandekerckhove J, Tuerlinckx F, Lee MD (2011). Hierarchical diffusion models for two-choice response times. Psychological Methods.

[CR64] Vehtari, A., Gabry, J., Magnusson, M., Yao, Y., Bürkner, P.-C., Paananen, T., & Gelman, A. (2020). loo: Efficient leave-one-out cross-validation and WAIC for Bayesian models.

[CR65] Vehtari A, Gelman A, Gabry J (2017). Practical bayesian model evaluation using leave-one-out cross-validation and WAIC. Statistics and Computing.

[CR66] Vierordt, K. (1868). Der Zeitsinn nach Versuchen. H. Laupp.

[CR67] Voss A, Nagler M, Lerche V (2013). Diffusion models in experimental psychology. Experimental Psychology.

[CR68] Wagenmakers E-J (2009). Methodological and empirical developments for the Ratcliff diffusion model of response times and accuracy. European Journal of Cognitive Psychology.

[CR69] Wiener M, Thompson JC, Coslett HB (2014). Continuous carryover of temporal context dissociates response bias from perceptual influence for duration. PloS One.

[CR70] Yeshurun Y, Carrasco M, Maloney LT (2008). Bias and sensitivity in two-interval forced choice procedures: Tests of the difference model. Vision Research.

[CR71] Zeigenfuse MD, Lee MD (2010). A general latent assignment approach for modeling psychological contaminants. Journal of Mathematical Psychology.

